# Certified Food Safety Management Systems Assessed through the Lenses of Food Safety Culture and *Locus* of Control: A Pilot Study

**DOI:** 10.3390/foods13172759

**Published:** 2024-08-29

**Authors:** Giada Forte, Simona Tornielli, Daniela Parini, Vera Lavelli

**Affiliations:** 1 Department of Food, Environmental and Nutritional Sciences, Università degli Studi di Milano, Via Celoria, 2, 20133 Milano, Italy; giada.forte@studenti.unimi.it; 2 Eurofin Food Assurance Italia, Via Bruno Buozzi, Milano 2, 20055 Vimodrone, Italy; simonatornielli@eurofins.com (S.T.); daniela.parini@virgilio.it (D.P.)

**Keywords:** food safety culture, *locus* of control, certified company, management system

## Abstract

The approach to ensure food safety (FS) has evolved, including the concept of FS culture, which has been shaped by both the legislation and the scientific literature. In this study, two companies that produce foods associated with potential risks of cross-contamination (gluten-free foods and frozen pastry, respectively) and are certified according to international voluntary FS standards, such as the British Retail Council Global Standard (BRC) and the International Featured Standards Food Version (IFS), were investigated to assess: (a) if the assessment of FS culture’s pillars can uncover unexpected critical areas; (b) if the scores of the FS culture’s pillars are related to personal traits, namely, age, seniority in the company and *locus* of control orientation, i.e., the beliefs that an event is the result of external factors (luck, destiny or superior beings), or the result of internal factors (human behavior). Questionnaires for the survey and the scoring system applied were selected from the literature. Results showed that all food handlers had an optimistic bias, which paradoxically could be the consequence of the rigorous application of hygienic procedures. The younger food handlers had significantly (*p* < 0.05) lower commitment than the older ones. Moreover, the segment of food handlers having an external *locus* orientation demonstrated weaker normative beliefs than those having an internal *locus* of control orientation. Results showed that the FS culture survey, which is related to the shared FS culture, could disclose unknown weakness in third-party certified companies, even if the well implemented principles of voluntary FS standards are aligned with the FS-culture pillars. Moreover, the segmentation of food handlers according to their age and the *locus* of control assessment could provide additional information on the individual orientation toward FS behavior. Hence these tools could assist the leaders in the management of the dynamic nature of human capital.

## 1. Introduction

The definition of “food safety” (FS) was provided by the Codex Alimentarius in 1969 and remained unchanged in the last revision of this document, as follows: “assurance that food will not cause adverse health effects to the consumer when it is prepared and/or eaten according to its intended use” [1]. This definition is well established, and to address this basic right for humans, the food law at the government level and the food management systems at the company level should be continuously developed and revised to include the emerging risks due to globalization, such as new ingredients, foods, and processing technologies [2], as well as to consider the impact of climate change on food contamination [3]. Moreover, changes in the economic and political scenario also have an impact on FS legislation. For instance, in 2007, when new states joined the European Union, 86 new regulations and 198 decisions were issued to harmonize food law among member countries [4,5,6,7].

In addition to the evolution of the above-described technical approach, a techno-managerial approach was introduced to ensure FS, which is focused on people working in the organizations and interacting with the technological system, thus creating a “socio-technical systems” [8]. This latter approach has led to the concept of “FS-culture”. The Global Food Safety Initiative (GFSI) has defined FS-culture as “shared values, beliefs and norms that affect mindset and behavior toward FS in, across and throughout an organization” [9]. The publication of this latter GFSI position paper in 2018 on FS-culture and the addition of FS-culture as a GFSI benchmark criterion has promoted different studies on this topic [10,11], which generally concluded that FS can be improved with the implementation of FS-culture assessment. Moreover, FS assessment was included in food hygiene guidelines, standards, and the EU regulation [1,12,13,14].

The interest in FS-culture is due to the concept that a strong FS-culture could be associated to low incidence of FS-related accidents and foodborne diseases. For instance, failures in the organizational food safety culture and lack of managerial engagement were associated with a multi-state listeriosis outbreak [15]. Moreover, the strategic importance of food safety culture was reinforced as it was found to be associated with a higher prevention and appraisal costs, lower failure costs, and lower overall quality costs [16]. Indeed, the FS-culture has emerged as a broader and more relevant concept than knowledge. Studies on FS-culture assessment were performed in food services, supermarkets, and food processing industries worldwide [17]. In all these studies, the FS-culture has been investigated by multiple dimensions, which commonly include eight pillars: leadership, communication, commitment, knowledge, FS management systems, risk perception, work environment, and normative beliefs [17]. A scoring system was developed for FS-culture pillars and the overall prevailing FS-culture, ranked as reactive, active or proactive [18,19]. Furthermore, this latter tool was applied to compare the results of periodic FS-culture assessments in a longitudinal study [20].

According to the GFSI position paper, the company’s overall values and mission affect the thinking of the individuals [9]. This approach is in line with one paradigm of FS voluntary standards, such as the British Retail Council Global Standard (BRC), the International Featured Standards Food Version (IFS), and the International Standard ISO 22000, which are widely accepted as tools that can guarantee a high level of FS and food law compliance. In fact, the implementation of ISO 22000 in food companies resulted in improved efficiency and food safety [21]. Moreover, the food retail industry drives the implementation of standards, such as IFS and BRC, all over the world to increase food safety [22]. Key strategies of these latter standards include the definition of a FS policy, internal communication of the FS policy, and the application of procedures and instructions to standardize knowledge and behavior, leading to the efficient implementation of tasks related to FS. Indeed, in a study covering 116 companies across developing and developed regions, it was found that the FS culture remarkably increased after the implementation of FS management systems in both developed and developing countries (*p* < 0.001) [23].

In addition to the efforts to standardize knowledge and behavior, according to the organizational sciences, the role of personality at work is also a relevant perspective to investigate job satisfaction and performance [24]. Hence, one challenging area of investigation is the related inter-individual differences in FS-culture pillar perception due to personal traits. In this context, job stress and burnout were shown to have a moderate effect on FS-culture, while motivation is a partial mediator between FS-culture and compliance and behavior [25].

Another trait of humans that might be related to FS-culture pillars is the orientation of the *locus* of control, which is instrumental to the organizational sciences [24]. The *locus* of control refers to how much individuals believe an event is the result of external factors (e.g., superior beings, luck, destiny) or the result of their own behavior, being conceptualized as external or internal, respectively. The *locus* of control orientation has been used in occupational safety [26,27,28,29]. Particularly, it was found that employees with greater internal safety *locus* of control orientations reported fewer occupational accidents and more safety performance at workplaces with respect to employees with an external *locus* of control. In high-hazard work environments, such as the mining industry, risk avoidance on near misses is enhanced as a miner’s *locus* of control increases [30]. The lessons learned from the investigation on occupational safety could be useful in analyzing the FS behavior of food handlers. Indeed, in the food context, the *locus* of internal control also seems to be related to the better adoption of FS practices than the external *locus* of control [31,32].

In the present study, two companies that produce foods associated with potential risks of cross-contamination (gluten-free foods and frozen pastry, respectively) and have aligned their management systems to the stringent requirements of international FS standards have been used as models to investigate the FS-culture and the *locus* of control among food handlers.

The aims were to verify the following hypothesis: (a) the FS-culture pillars’ assessment can uncover shared critical areas; (b) the FS-culture pillars can uncover individual weakness, i.e., their scores are related to personal traits (seniority in the company, *locus* of control); (c) these assessments can consequently represent an input for the continuous improvement of the management system.

## 2. Materials and Methods

### 2.1. Case Studies

Two model food manufacturing companies, A and B, located in northern Italy, were selected for the study. Company A (200 employees) produces frozen pastries to be sold to retailers and the hotel-restaurant-catering network. It has BRC, IFS food, UTZ (“UTZ” means “good” in the Mayan language, and the UTZ certification is a program to promote sustainability within the coffee, cocoa, tea, and hazelnut supply chains), Halal (“Halal” is the Arabic word for “permissible” and Halal certification ensures that foods have been processed in accordance with Islamic requirements), and free-range eggs certificates. Food handlers of company A involved in the survey (154 responders) were female (63%), males (30%) or not declared (7%); their education level was a tertiary school (92), secondary school (37), Bachelor or Master degree (18), or not declared (7). They were grouped according to age: <30 years (39), from 31 to 40 years (55) or >40 years (60); seniority in the food company: <5 years (59); from 6 to 10 years (49); >10 years (46); seniority in the food sector: <5 years (38), from 6 to 10 years (41), from 11 to 15 years (35), >16 years (40). All food handlers take part in FS training at least once a year.

Company B (150 employees) produces conventional and gluten-free pastries to be sold to retailers and the hotel-restaurant-catering network. It has BRC and IFS food certificates. Food handlers of company B involved in the survey (90 responders) were female (38%) and males (52%), while the remaining 10% did not declare their gender; their education level was a tertiary school (58), secondary school (30), Bachelor or Master degree (2). They were grouped according to age: <40 years (25), from 41 to 50 years (24) or >50 years (41); seniority in the food company: <10 years (38), from 11 to 25 years (33), >26 years (19); seniority in the food sector: <5 years (11); from 6 to 10 years (14); from 11 to 15 years (13); from 16 to 20 years (12), from 21 to 25 years (11), >26 years (29). All food handlers take part in FS training at least once a year.

### 2.2. FS-Culture and Locus of Control Questionnaires

The assessment of FS-culture was performed through the questionnaire developed previously [18], using the therein proposed scoring and classification system. In brief, the FS-culture pillars included: “leadership” (5 items), “communication” (4 items), “commitment” (16 items), “management system” (6 items), “work environment” (3 items), and “work pressure and normative beliefs” (6 items) assessed by a five-points Likert scale (classification: mean from 1.0 to 2.5 = reactive, from 2.6 to 4.0 = active, from 4.1 to 5 = proactive); “knowledge” (10 items) assessed by score 1 for correct answers and score 0 for wrong and “I do not know” answers (classification: mean from 0.0 to 5.0 = reactive; from 5.1 to 7.5 = active; from 7.6 to 10.0 = proactive); and “risk perception” (4 items), assessed by a scale from −3 to +3 (classification: mean from −3.0 to −1.1 = reactive; from −1.0 to +1.9 = active; from +2.0 to +3 = proactive). The *locus* of control (8 items) was assessed according to a questionnaire previously developed [32], using a couple of alternative statements, either for internal orientation or for external orientation. Score 1 was assigned to the internal orientation answers, and score 0 was assigned to the external orientation answers.

### 2.3. Statistical Analysis

Internal consistency, i.e., Cronbach’s α [33], was calculated for the continuous pillars. A paired Student’s t-test was performed to identify the optimistic bias of food handlers by comparing the answers to questions 1 and 2 and the answers to questions 2 and 3 in the risk perception section [34]. Optimistic bias was identified for *p* < 0.05. One-way ANOVA was applied to evaluate differences in the scores due to age, seniority in the company, and seniority in the food sector of the participants, using the least significant difference (LSD) as a multiple-range test; differences were considered significant for *p* < 0.05. To this aim, participants were segmented as described in the previous paragraph. Pearson correlations were performed between the *locus* of control scores and the FS-culture pillars scores and were considered to be significant for *p* < 0.05. The Statgraphics (STCC Inc.; Rockville, MD, USA) 5.1 software was used.

## 3. Results and Discussion

### 3.1. Shared FS-Culture, Risk Perception, and Optimistic Bias

Table 1 presents an overview of the FS-culture scores observed for companies A and B. Overall, the pillars had a good internal consistency, from 0.74 to 0.89. As expected, both companies, which are certified according to international FS standards, achieved “active” or “proactive” scores for most of the pillars. On the other hand, the score of risk perception was “reactive”. In a previous study, certified companies achieved higher average scores than non-certified companies in FS-culture and proved to have overall high FS-culture, but the dimensions assessed were different from those considered here, namely, knowledge, business priorities, and FS legislation [35].

An insight on risk perception is shown in Table 2. Using a scale between −3 (low risk) and +3 (high risk) the average answer to question R 4, “If a consumer eats contaminated food, what is the risk of a foodborne disease being severe or lethal to him/her?” was 0.1 ± 1.5 and 0.38 ± 2.1 for the food handlers of company A and B, respectively. It is worth considering that both companies are involved in the processing of foods that are associated with potential risks of contamination leading to severe disease, such as, for instance, gluten contamination of foods for celiac subjects and microbial contamination since pastries have a neutral pH and a high water activity. Hence, food handlers showed a low perception of the severity of the risk of food contamination. Moreover, the food handlers of company A scored −2.2 ± 1.4, −1.4 ± 1.9, and −1.8 ± 1.5 on the likelihood occurrence of a foodborne disease as a consequence of their own work, the work of other food handlers in other companies, and the work of other employees in the same company, respectively (Table 2). Similarly, the food handlers of company B scored −2.1 ± 1.6, −1.3 ± 2.1, and −1.7 ± 1.7 on the likelihood of occurrence of a foodborne disease as a consequence of their own work, the work of other food handlers in other food companies, and the work of other employees in the same company, respectively (Table 2). Hence, the food handles of both companies revealed an optimistic bias in relation to the work of other food handlers in other companies (question 1 versus question 2: *p* < 0.001 for company A and *p* < 0.01 for company B). The food handlers of company A, but not those of company B, also showed an optimistic bias in relation to the other employees in the same company (question 2 versus question 3: *p* < 0.02).

Previous studies have shown that individuals tend to underestimate different kinds of personal risks [36]. This phenomenon is referred to as optimistic bias and is conceptualized as the belief of individuals that they are less prone to experience negative events than their peers. A food handler with an optimistic bias has a positive outlook on the risks. Three main reasons can explain the optimistic bias. First, the repetition of the same tasks (routine) gives confidence to food handles and favors optimistic bias [34]. Secondly, the belief that lack of experience with a hazard in the past is protective against experience in the future also contributes to the optimistic bias [37]. For instance, optimistic bias regarding food contamination was found to be higher in people who had not experienced food poisoning than in people who had experienced Salmonella food poisoning, despite no differences being found in knowledge [38]. It is important to specify that in the aforementioned study, Salmonella poisoning was chosen not only for its importance as a food pathogen but also because the majority of Salmonella poisoning cases are sporadic rather than part of a recognized general outbreak [38]. The work context, such as adequate structures and procedures and frequent training about food handling, favors the optimistic bias. Indeed, it was observed that food handlers from schools and hospitals, where hygienic rules are more severe, presented higher optimistic bias levels than food handlers from street food vendors and restaurants [34]. Finally, the optimistic bias is also linked to self-esteem, namely the belief of an individual to be able to successfully achieve a specific goal [37].

Results from the current study showed that there were no significant differences in the scores of risk perception as related to age and seniority of food handlers in the food companies A and B (for instance, for company A, the average scores for risk perception were: −1.29, −1.20, and −1.37 and for company B the average scores were −1.06, −1.06, and −1.26 for the age groups 1, 2, and 3, respectively); hence, the routine is not likely the main cause for the observed optimistic bias. Both companies have not experienced severe food contamination and are certified with respect to the IFS and BRC standards that require restrictive hygienic rules and frequent training, which could explain the occurrence of optimistic bias, as observed previously [34]. Finally, the food handlers of companies A and B showed a high perception of food commitment, achieving a proactive score that can indicate a self-esteem perception, which is also related to the optimistic bias [37].

Based on these considerations, optimistic bias has a positive connotation when it is considered to evaluate past events since it suggests that hygienic procedures were set, training was performed, no harmful events have occurred, and food handlers have self-esteem. However, optimistic bias raises concern when it is considered to forecast future events because it may cause progressive misapplication of the rules and an inaccurate estimate of the probability of occurrence of hazards. This suggests that one paradigm of the risk analysis, i.e., the likelihood of occurrence of a specific hazard, can be calculated by considering the historical occurrence of hazards should be revised. Indeed, prevision models to calculate the risk level are based on the frequency of contamination events [39], while a comprehensive assessment of the risk level should also consider the evolving risk perception of the food handlers. Hence, the optimistic bias could be considered as a baseline for the assessment of the probability of occurrence of food hazards in risk analysis.

### 3.2. Individual Level of FS-Culture

#### 3.2.1. Age

The commitment scores were significantly affected by the age of food handlers: the higher the age, the higher the commitment score (Table 3). It was explained that the commitment includes three separable components reflecting (a) a desire (affective commitment), (b) a need (continuance commitment), and (c) an obligation (normative commitment) to maintain employment in an organization. All the components of commitment were affected by food handlers’ age [40]. The commitment was found to be a strong antecedent to employee performance [41]. In the context of food companies, FS commitment was found to have a stronger influence on food handler behavior than knowledge [42]. One major driver of food commitment was found to be the leadership style. Transactional leadership (focusing on instruction, training, and food handlers’ participation in decision-making) and transformational leadership (focusing on improving motivation, tuning vision, and enhancing creativity) increase food handlers’ job satisfaction and commitment and consequently improve food handlers’ hygienic practices. Then, food handlers with high commitment adhere to hygienic practices not because of being monitored and supervised but because they believe in and are committed to complying [43]. Accordingly, in the current study, for the food handlers of company B, the lower the commitment, the lower the perceptions of both leadership and communication. For company A, however, there were no differences in the perception of leadership and communication in different age groups. On the other hand, as shown in Table 3, for both companies, a personal trait such as age strongly affected the three components of the commitment. This finding suggests that the management of the human capital should combine food handlers of different ages to balance the commitment and hence improve the efficacy of teamwork. Moreover, despite the annual repetition of training activities, in both companies, there were no differences in knowledge among food handlers of different ages. Similarly, in a large survey involving food handlers in the food serving sector, no differences in knowledge were observed due to their age [44]. On the other hand, a survey regarding online FS courses revealed that the training efficacy was higher for older food workers than for younger food workers, probably due to higher self-regulation [45]. 

#### 3.2.2. Seniority in the Food Company

As a general rule, the seniority of food handlers in the food company did not explain the variance of the scores of the FS-culture pillars, i.e., the group of food handlers with seniority less than 5 years did not rank the items differently with respect to the food handlers with higher seniority, up to 26 years or more. This result suggests that in the certified companies A and B, new personnel is leveled with senior personnel relatively rapidly, probably through the application of a shared policy and standard procedures.

The only exception was the continuous commitment of food handlers, which increased as the seniority increased (*p* = 0.08 for company A and *p* = 0.02 for company B). On the other hand, the continuous commitment was most significantly affected by the age of food handlers (*p* = 0.01 for company A and *p* = 0.0015 for company B), as discussed in the previous paragraph.

#### 3.2.3. *Locus* of Control

Using a scale from 0 (full external *locus*) to 8 (full internal *locus*), the food handles of company A had a *locus* of 5.7 ± 1.5, while those of company B had a *locus* of 5.6 ± 1.8. The statement that addressed the responsibility for FS (i.e., “We have little control over the food we prepare and over the things that happen to them”) was found to be true for 30% of food handles of company A and 36% of food handles of company B. Hence, one third of food handlers in each company were not aware of the impact of their work on FS. The *locus* of control was correlated with the sum of the scores of all FS-culture pillars (*p* < 0.05), excluding risk perception. However, as discussed before, the results for risk perception are biased. The highest correlations were observed between the *locus* of control and normative beliefs (generally *p* < 0.05, Table 4).

The observed correlation between the *locus* of control and normative beliefs suggests that there is a relationship between the *locus* of control and the perception of the rules posed by the leadership, the health surveillance authorities, the customers, and the work partners. This result is consistent with a previous study that reported that the psychologist’s notion of ‘*locus* of control’ is very closed to responsibility [46]. In fact, studying the pro-environmental behavior of individuals, it was observed that people who do not act pro-environmentally feel they should not have to take responsibility for it [46]. Previous meta-analysis showed that the *locus* of control is correlated to job satisfaction (*p* < 0.05), including satisfaction with job supervisors and job co-workers [24]. This latter correlation, combined with that observed in the present study, suggests that normative beliefs and job satisfaction could also be closely related. Specifically, job satisfaction could provide a basis for high perception of the rules and judgments of the leadership and the co-workers.

Considering the positive outcomes of the internal *locus*, the identification of the factors that affect the *locus* of control orientation is crucial. It was ascertained that the orientation of *locus* of control was related to personal traits and religious beliefs [32]. In a previous study, cognitive illusions emerged among food handlers, which were related to their religious beliefs. In particular, the believe that the use of objects, such as chains and rings, related to religion provides protection from different harms, including food contamination [32]. In the same study, it was reported that one worker affirmed that contamination is due to the origin of the products and not because of handling, clearly revealing the belief that superior beings are responsible for events [32]. On the other hand, context-related factors, especially training, can also influence the *locus* of control. Indeed, specific interventions such as active learning methods centered on dialogue and problem-solving as key elements of the learning process seemed to orient the *locus* of control internally [47,48]. Hence, it is advisable to develop strategies for the dissemination of FS culture, even in companies that are certified according to FS standards, as already observed in a previous study [49]. Moreover, the integration of *locus* of control assessment can offer new insights into a poorly explored area of food safety management systems. Indeed, uncontrollable personal events and beliefs can affect the *locus* of control of the food handlers and potentially impact rule adoptions; at the same time, the context can contribute to the *locus* of control orientation. Even though the balance between these interplaying factors cannot be fully controlled, the *locus* of control should not be minimized or ignored, and efforts should be made by the leadership to favor its internal orientation.

## 4. Practical Implications, Limitations, and Future Research

The practical implications of this study are that the assessment of FS-culture with a validated questionnaire can disclose unknown weaknesses in certified food companies and that the individual variability poses a challenge to the purpose of standardization, which is the basis of FS voluntary standards and third-party certification.

The survey involved only two companies, which were similar in terms of production sector (high-risk foods) and geographical location. Future studies may assess different food contexts to evaluate how personal traits, including those not considered here, such as, *in primis*, ethnicity, education, and psycho-social well-being, affect FS. Moreover, periodic assessments to monitor changes in FS culture and individual traits over time, including control groups from companies with and without certified FS management systems, could provide additional comparative insights. By capturing the relevant differences among individuals, it would be challenging to balance the purpose of standardizing the risk prevention procedures with the unavoidable human variability, thus designing a holistic model of FS management.

## 5. Conclusions

The case studies considered, which are food companies certified according to international FS standards, were found to achieve, on average, a high FS-culture score, but three potentially dangerous orientations emerged through the lenses of FS-culture and *locus* of control questionnaires.

Firstly, all food handlers revealed an optimistic bias, which paradoxically could be the consequence of the rigorous application of hygienic procedures. Secondly, the younger food handlers had lower commitment than the older ones, which could be related to less efficient FS behavior. Finally, the segment of food handlers having an external *locus* orientation demonstrated weaker normative beliefs that could uncover a low sense of responsibility.

Hence, the FS-culture proved to be an interesting tool for investigating both shared and individual orientation toward FS behavior. Moreover, the combination of FS-culture assessment with cognitive psychology investigation approaches, such as the assessment of the *locus* of control, which already proved to be instrumental in the context of occupational safety, would provide a better insight into food handlers’ behavioral intentions. The results of these assessments could assist the leaders not only in the design of training activities but, more widely, in risk analysis and the management of human capital. As a general conclusion, since the human factor is dynamically modified not only by personal events but also by the organization’s environment, a periodical application of FS-culture and *locus* of control assessments could track the evolution of the FS management system performance.

## Figures and Tables

**Table 1:** Scores (mean ± SD) and classification of the FS-culture pillars for companies A and B.

	Company A		Company B	
Leadership ^1^	4.1 ± 1.1	3 proactive	3.5 ± 1.3	2 active
Communication ^1^	3.9 ± 1.2	2 active	3.6 ± 1.3	2 active
Knowledge ^2^	6.7	2 active	6	2 active
Commitment ^1^	3.5 ± 1.4	2 active	3.3 ± 1.6	2 active
Risk perception ^3^	−1.4 ± 1.8	1 reactive	−1.2 ± 2.1	1 reactive
Work pressure and normative beliefs ^1^	4.1 ± 1.3	3 proactive	4.0 ± 1.4	2 active
Work environment ^1^	4.6 ± 0.8	3 proactive	3.8 ± 1.3	2 active
Management system ^1^	3.4 ± 1.4	2 active	3.2 ± 1.4	2 active

^1^ Pillars assessed by a 5-point Likert scale. Classification: mean between 1.0 and 2.5—reactive; from 2.6 to 4.0—active; from 4.1 to 5—proactive. ^2^ Pillar assessed by score 0 to 10; score < 5.0 insufficient, from 5.1–7.5 medium, ≥7.6 sufficient. Classification: mean between 0.0 and 5.0—reactive; from 5.1 to 7.5—active; from 7.6 to 10.0—proactive. ^3^ Pillar was assessed using a scale from −3 to +3. Classification: mean between −3.0 and −1.1—reactive; from −1.0 to +1.9—active; from +2.0 to +3—proactive [18].

**Table 2:** Scores (mean ± SD) for the individual risk-related questions, *p*-values for optimistic bias 1 (OB 1, question 1 × question 2) and optimistic bias 2 (OB 2, question 3 x question 2), and overall score for FS commitment assigned by the food handlers of the companies A and B.

	Company A		Company B	
Risk-related questions ^1,2^				
- If a consumer eats contaminated food, what is the risk of a foodborne disease being severe or lethal to him/her? (question R 4)	0.1 ± 1.5		0.38 ± 2.1	
- What is the consumer’s likelihood of presenting abdominal pain and/or vomiting (foodborne disease) after eating a meal prepared by …				
- … you (question R 2)	−2.2 ± 1.4		−2.1 ± 1.6	
- … a food handler similar to you (with similar age and who participated in the same amount of training as you), but who works for another establishment (question R 1)	−1.4 ± 1.9	*p* = 0.001 (OB 1)	−1.3 ± 2.1	*p* = 0.01 (OB 1)
- … one of your colleagues (food handler who works at the same establishment) (question R 3)	−1.8 ± 1.5	*p* = 0.02 (OB 2)	−1.7 ± 1.7	*p* = 0.09 (no OB 2)
Commitment to FS ^1,3^	4.7 ± 0.8		4.5 ± 0.9	

^1^ The questions were taken from Zanin et al., 2021 [18]. ^2^ Questions were assessed on a scale from −3 to +3. ^3^ Questions assessed by a 5-point Likert scale and then averaged to obtain the overall score.

**Table 3:** Overall scores (mean ± SD) for the affective commitment, continuous commitment, normative commitment, leadership, and communication and scores for the individual questions related to each component of the commitment assigned by food handlers of companies A and B as a function of age.

	Company A			Company B		
		Age Groups ^1^			Age Groups ^1^	
	≤30	31–40	>40	≤40	41–50	>50
Affective commitment ^2,3^	3.3 ^A^ ± 1.1	3.6 ^AB^ ± 0.9	3.8 ^B^ ± 1.0	2.6 ^A^ ± 1.2	3.3 ^AB^ ± 0.9	3.3 ^B^ ± 1.3
Continuous commitment ^2,3^	2.5 ^A^ ± 1.0	2.7 ^A^ ± 1.2	3.2 ^B^ ± 1.2	2.3 ^A^ ± 0.9	2.6 ^A^ ± 1.2	3.3 ^B^ ± 1.3
Normative commitment ^2,3^	2.7 ^A^ ± 1.0	2.8 ^A^ ± 1.1	3.3 ^B^ ± 1.0	3.2 ^A^ ± 1.1	3.9 ^B^ ± 0.9	3.7 ^AB^ ± 1.1
Leadership ^2,3^	4.1 ± 1.0	4.0 ± 0.8	4.0 ± 0.9	3.1 ^A^ ± 1.3	3.4 ^AB^ ± 1.1	3.8 ^B^ ± 1.0
Communication ^2,3^	4.0 ± 1.1	3.9 ± 0.9	3.8 ± 1.0	3.2 ^A^ ± 1.1	3.9 ^B^ ± 0.9	3.7 ^AB^ ± 1.1
Affective commitment-related questions						
- I feel like the company’s problems are mine.	2.7 ^A^ ± 1.4	3.2 ^AB^ ± 1.1	3.4 ^B^ ± 1.4	2.4 ± 1.3	2.8 ± 1.3	3.0 ± 1.4
- This company has great personal meaning for me.	3.1 ^A^ ± 1.5	3.6 ^B^ ± 1.2	3.7 ^B^ ± 1.4	2.6 ^A^ ± 1.3	3.4 ^B^ ± 1.2	3.3 ^B^ ± 1.5
- This company deserves my loyalty.	3.7 ± 1.5	3.9 ± 1.2	4.0 ± 1.6	3.0 ± 1.5	3.5 ± 1.2	3.5 ± 1.6
- Working at this company is both a need and a wish.	3.5 ± 1.6	3.6 ± 1.2	3.9 ± 1.2	2.7 ± 1.4	3.3 ± 1.5	3.3 ± 1.3
- I would be pleased to dedicate the rest of my career to this company.	3.2 ± 1.5	3.4 ± 1.2	3.8 ± 1.6	2.5 ^A^ ± 1.4	3.3 ^AB^ ± 1.4	3.4 ^B^ ± 1.6
Normative commitment-related questions						
- I would not quit my job right now because I have a moral obligation to the staff around.	2.3 ± 1.2	2.3 ± 1.1	2.9 ± 1.2	2. 2 ± 1.2	2.0 ± 1.2	2.4 ± 1.4
- I would feel guilty if I quit my job right now.	2.7 ± 1.4	2.9 ± 1.2	3.1 ± 1.5	2.3 ± 1.4	2.3 ± 1.6	2.6 ± 1.7
- Even if it was an advantage for me, I feel like it is not correct to quit my job now.	2.1 ^A^ ± 1.3	2.6 ^B^ ± 1.7	3.0 ^B^ ± 1.3	2.1 ± 1.1	2.0 ± 1.3	2.5 ± 1.6
Continuous commitment-realated questions						
- If I decide to quit my job now, my life would be really disrupted.	2.5 ^A^ ± 1.3	2.8 ^AB^ ± 1.3	3.3 ^B^ ± 1.3	2.5 ± 1.3	2.2 ± 1.1	2.7 ± 1.6
- I think I would have a few options if I quit my job now.	2.1 ^A^ ± 1.2	2.5 ^A^ ± 1.3	3.2 ^B^ ± 1.3	2.0 ^A^ ± 1.3	2.4 ^A^ ± 1.6	3.7 ^B^ ± 1.4
- Even if I wanted to, it would be really hard for me to quit my job now.	2.8 ± 1.3	3.0 ± 1.4	3.0 ± 1.3	2.3 ^A^ ± 1.30	3.1 ^AB^ ± 1.6	3.7 ^B^ ± 1.6

^1^ Different letters in a row indicate significant differences among age groups within the same company (*p* ≤ 0.05). ^2^ The questions were taken from Zanin et al., 2021 [18]. Questions were assessed by a 5-point Likert scale. ^3^ The individual questions of the same pillar or pillar component were averaged to obtain the overall score.

**Table 4:** Correlation between the normative beliefs scores and the *locus* of control scores for food handlers of companies A and B.

	Company A		Company B	
	Slope	R	Slope	R
Sum of normative beliefs scores	0.71 ± 0.14	0.38 **	0.65 ± 0.15	0.42 **
Normative beliefs related questions ^1^				
- My boss thinks that I need to follow good practices for food handling in all my tasks.	0.55 ± 0.13	0.35 **	0.68 ± 0.16	0.44 **
- My work partners think that I need to follow the hygiene norms in all my tasks.	0.35 ± 0.11	0.25 **	0.37 ± 0.13	0.31 *
- The health surveillance authorities think that I need to follow the hygiene norms in all my tasks.	0.65 ± 0.12	0.14 **	0.57 ± 0.14	0.42 **
- The clients of this establishment think that I need to follow the hygiene norms in all my tasks.	0.70 ± 0.14	0.38 **	0.49 ± 0.15	0.35 *

^1^ The questions were taken from Zanin et al., 2021 [18]. * = *p* < 0.05; ** = *p* < 0.01.

## Data Availability

The original contributions presented in the study are included in the article, further inquiries can be directed to the corresponding author.
